# Supplementary material for: Arabidopsis root apical meristem adaptation to an osmotic gradient condition: an integrated approach from cell expansion to gene expression
Source: Front Plant Sci. 2024 Nov 7;15:1465219. doi: 10.3389/fpls.2024.1465219 (PMC11579709; doi:10.3389/fpls.2024.1465219)
Supplement: Supplementary file 1 [file DataSheet1.pdf]

Supplementary Material

1 Supplementary Figures and Tables

1.1 Supplementary Figures

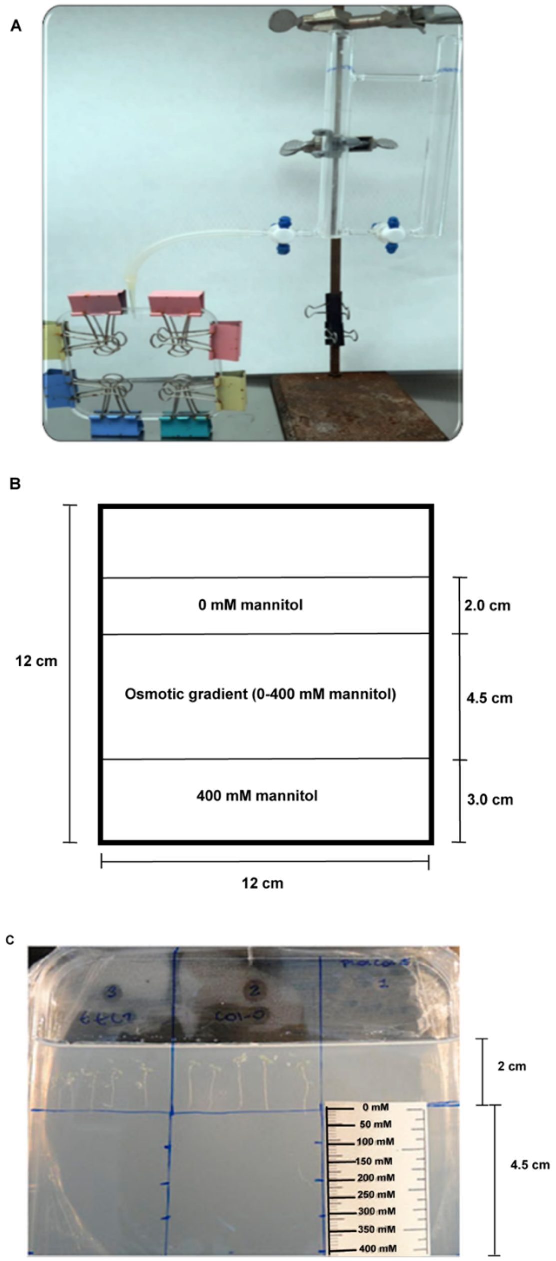

Supplemental Figure 1. System used to establish the osmotic gradient. **A.** Gradient maker. **B.** Vertical acrylic plate. **C.** Representative schematic of a Petri dish containing the osmotic gradient.

Sequentially, 1 block of medium with 400 mM mannitol (3.0 cm), 1 block of medium with osmotic gradient (4.5 cm), and 1 block of medium without mannitol (2.0 cm) were placed.

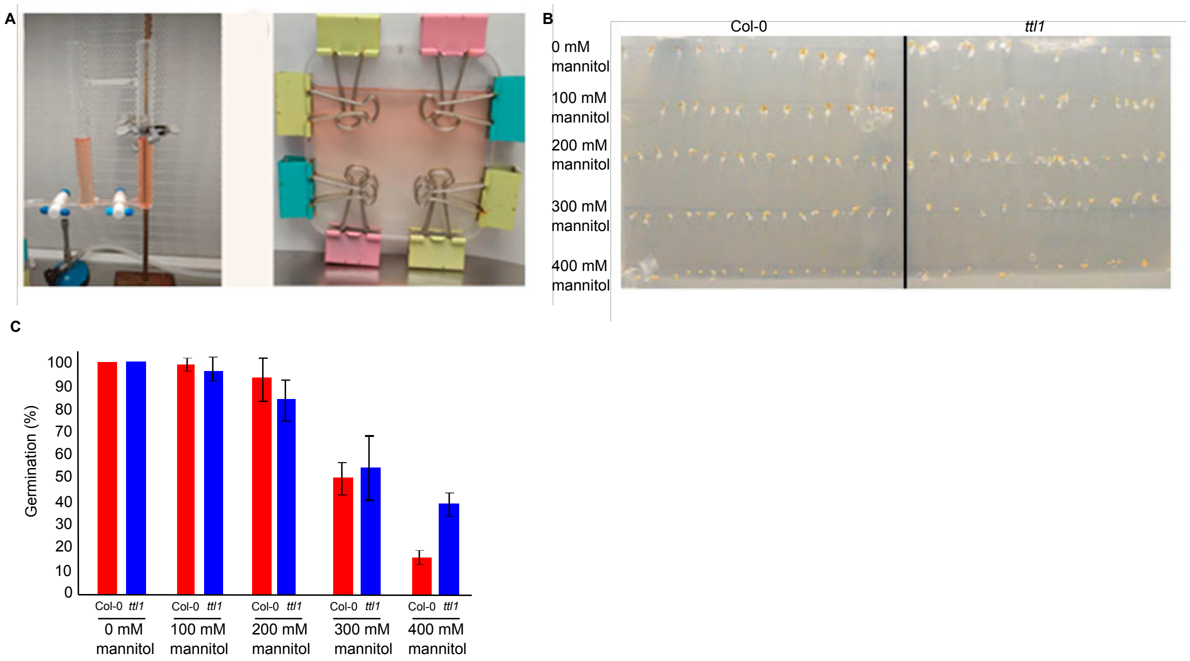

Supplemental Figure 2. Image depicting how seedlings were planted in the osmotic gradient system. Seedlings are placed in 0 mM mannitol, and the root tips are positioned at the entry to the gradient. The ruler is shown, correlating mannitol concentrations with osmotic potentials measured using the OSMOMAT 030 osmometer model (Gonotech, Berlin, Germany).

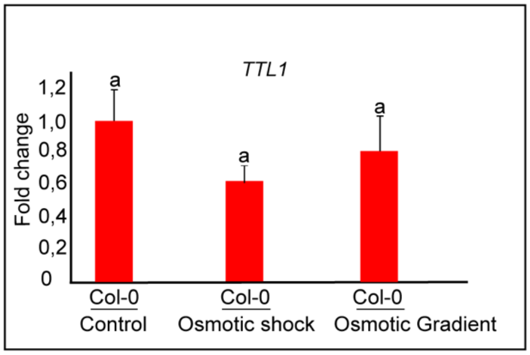

Supplemental Figure 3. *TTL1* expression levels in roots of Col-0 grown in control, osmotic shock and osmotic gradient conditions. Data are presented as means ± standard deviation (SD). Three biological replicates and two technical replicates were considered, and gene expression is relative

to *CYTOCHROME C OXIDASE RELATED*. Different letters indicate statistically significant differences (Tukey test P value < 0.05).

1.2 Supplementary Tables

Supplemental Table 1: PCA Correlation Values

Gene	PC1	PC2
AHA1	**0,86**	-0,13
AHA2	**0,89**	-0,26
BES1	-0,64	-0,47
CESA1	**0,71**	-0,47
CESA3	**0,88**	0,14
CESA6	**0,71**	-0,38
CSLA9	0,18	**-0,81**
COBRA	0,05	**-0,9**
CPD	-0,53	**-0,76**
CYCD3;1	**0,84**	0,04
DWF4	-0,42	**-0,81**
EXPA1	0,27	0,1
PLL12	**0,8**	-0,09
TTL3	**0,9**	-0,03

Supplemental Table 2. Two-way ANOVA analysis followed by a mean comparison test was used to identify significant differences (P value < 0.05) between root growth rates of Col-0 and *ttl1* in osmotic shock and osmotic gradient using R Software. A. Two-way ANOVA. B. Mean comparison test in osmotic gradient. C. Mean comparison test in osmotic shock.

```
###  Osmotic Gradient #################

mod.1_gr1 <- lm ( b1 ~ genotype + media + genotype * media, data = df.gradient )

Analysis of Variance Table

Response: b1
                 Df  Sum Sq  Mean Sq F value    Pr(>F)
genotype          1 0.03426 0.034257  3.9045   0.05055 .
media             3 0.74667 0.248890 28.3678 8.363e-14 ***
genotype:media    3 0.02037 0.006789  0.7738   0.51095
Residuals       115 1.00897 0.008774
---
Signif. codes:  0 ‘***’ 0.001 ‘**’ 0.01 ‘*’ 0.05 ‘.’ 0.1 ‘ ’ 1
```

```
###  Osmotic Shock #################
anova (mod.1_shock)
Analysis of Variance Table

Response: b1
              Df Sum Sq Mean Sq  F value  Pr(>F)
genotype        1 0.0252 0.02518   4.1689 0.04276 *
media           2 3.3973 1.69866 281.2338 < 2e-16 ***
genotype:media   2 0.0086 0.00429   0.7109 0.49269
Residuals      165 0.9966 0.00604
---
Signif. codes:  0 ‘***’ 0.001 ‘**’ 0.01 ‘*’ 0.05 ‘.’ 0.1 ‘ ’ 1
```

B. Mean comparison test in osmotic gradient.

contrast	estimate	SE	df	t.ratio	p.value
**(Col-0 0_50mM) - ttl1 0_50mM**	0.0714	0.0342	115	2.0878	0.4289
**(Col-0 0_50mM) - (Col-0 200_300mM)**	0.0307	0.0354	115	0.8661	0.9884
**(Col-0 0_50mM) - ttl1 200_300mM**	0.0780	0.0362	115	2.1526	0.3882
**(Col-0 0_50mM) - (Col-0 300_400mM)**	0.0664	0.0382	115	1.7384	0.6622
**(Col-0 0_50mM) - ttl1 300_400mM**	0.1431	0.0429	115	3.3383	0.0243
**(Col-0 0_50mM) - (Col-0 Control)**	-0.1024	0.0308	115	-3.3243	0.0254
**(Col-0 0_50mM) - ttl1 Control**	-0.0869	0.0302	115	-2.8816	0.0859
**ttl1 0_50mM - (Col-0 200_300mM)**	-0.0406	0.0355	115	-1.1457	0.9450
**ttl1 0_50mM - ttl1 200_300mM**	0.0067	0.0363	115	0.1842	0.9999
**ttl1 0_50mM - (Col-0 300_400mM)**	-0.0049	0.0382	115	-0.1289	0.9999
**ttl1 0_50mM - ttl1 300_400mM**	0.0717	0.0429	115	1.6728	0.7045
**ttl1 0_50mM - (Col-0 Control)**	-0.1739	0.0308	115	-5.6406	3.3817 e-6
**ttl1 0_50mM - ttl1 Control**	-0.1583	0.0302	115	-5.2490	1.9192 e-5
**(Col-0 200_300mM) - ttl1 200_300mM**	0.0474	0.0375	115	1.2628	0.9104
**(Col-0 200_300mM) - (Col-0 300_400mM)**	0.0357	0.0394	115	0.9070	0.9849
**(Col-0 200_300mM) - ttl1 300_400mM**	0.1123	0.0439	115	2.5594	0.1819
**(Col-0 200_300mM) - (Col-0 Control)**	-0.1332	0.0323	115	-4.1304	0.0017
**(Col-0 200_300mM) - ttl1 Control**	-0.1176	0.0316	115	-3.7212	0.0072
**ttl1 200_300mM - (Col-0 300_400mM)**	-0.0116	0.0401	115	-0.2896	0.9999
**ttl1 200_300mM - ttl1 300_400mM**	0.0650	0.0445	115	1.4599	0.8267
**ttl1 200_300mM - (Col-0 Control)**	-0.1805	0.0331	115	-5.4529	7.8417 e-6
**ttl1 200_300mM - ttl1 Control**	-0.1650	0.0325	115	-5.0778	4.0054 e-5

**(Col-0 300_400mM) - ttl1 300_400mM**	0.0766	0.0462	115	1.66059	0.7122
**(Col-0 300_400mM) - (Col-0 Control)**	-0.1689	0.0353	115	-4.7927	1.3163 e-4
**(Col-0 300_400mM) - ttl1 Control**	-0.1534	0.0347	115	-4.4240	5.7103 e-4
**ttl1 300_400mM - (Col-0 Control)**	-0.2456	0.0402	115	-6.1045	3.9644 e-7
**ttl1 300_400mM - ttl1 Control**	-0.2300	0.0397	115	-5.7907	1.7065 e-6
**(Col-0 Control) - ttl1 Control**	0.0155	0.0263	115	0.5924	0.9989

C. Mean comparison test in osmotic shock.

contrast	estimate	SE	df	t.ratio	p.value
**(Col-0 0.3M) - ttl1 0.3M**	0.0497	0.0196	165	2.5371	0.1195
**(Col-0 0.3M) - (Col-0 0.4M)**	0.1457	0.0202	165	7.1980	3.0669 e-10
**(Col-0 0.3M) - ttl1 0.4M**	0.1738	0.0204	165	8.5099	1.7131 e-13
**(Col-0 0.3M) - (Col-0 Control)**	-0.2007	0.0213	165	-9.4322	1.6542 e-14
**(Col-0 0.3M) - ttl1 Control**	-0.1852	0.0206	165	-8.9825	2.5091 e-14
**ttl1 0.3M - (Col-0 0.4M)**	0.0959	0.0198	165	4.8499	4.1408 e-5
**ttl1 0.3M - ttl1 0.4M**	0.1240	0.0199	165	6.2121	6.1276 e-8
**ttl1 0.3M - (Col-0 Control)**	-0.2505	0.0208	165	-12.0145	0
**ttl1 0.3M - ttl1 Control**	-0.2349	0.0202	165	-11.6487	0
**(Col-0 0.4M) - ttl1 0.4M**	0.0281	0.0206	165	1.3651	0.7477
**(Col-0 0.4M) - (Col-0 Control)**	-0.3464	0.0214	165	-16.1535	0
**(Col-0 0.4M) - ttl1 Control**	-0.3309	0.0208	165	-15.9192	0
**ttl1 0.4M - (Col-0 Control)**	-0.3745	0.0216	165	-17.3247	0
**ttl1 0.4M - ttl1 Control**	-0.3589	0.0209	165	-17.1248	0
**(Col-0 Control) - ttl1 Control**	0.0157	0.0218	165	0.7139	0.9800

Supplemental Table 3. Primer's sequences used in RT-qPCR analysis in this study.

**Gene to amplify**	**Primer sequence**
Actin 2 (AT3G18780)	Fw: 5' - CGT ACA ACC GGT ATT GTG CTG GAT -3'
	Rv: 5' – GCT TGG TGC AAG TGC TGT GAT TTC- 3'
Cytochrome C oxidase related (AT4G37830)	Fw: 5'- GCG ATT GTA CGT TCA GCT CTT TC-3'
	Rv: 5'- GTG CTC TTT GTT GTG CTT CAC C-3'
CESA1 (AT4G32410)	Fw: 5'- TCA TCA TAC CCG AGA TAA GCA AC-3'

	Rv: 5'- ACG ACT GGT AGC CAA CTG TTT AC-3'
CESA3 (AT5G05170)	Fw: 5'- CGT GCT GAC AGG ACC AGT ATT-3'
	Rv: 5'- CTT ACT CGT GGG AAG GGA GAG G-3'
CESA6 (AT5G64740)	Fw: 5'- ACA GCA CAG AAA GTG CCT GAG-3'
	Rv: 5'- GGA GCA TTT GAT AGA ACC CCA-3'
TTL1 (AT1G53300)	Fw: 5'- GCT AGC CAA ATC GAT CCA AG-3'
	Rv: 5'-AGC TCC CCA TCT TTC CAT CT-3'
TTL3 (AT2G42580)	Fw:5'- ATG GTG GAT GTG GAG GAG AG-3'
	Rv: 5'- AGC AGA GCC AAA CTC CAA AA-3'
PECTATE LYASE12 (AT5G04310)	Fw: 5'-CGG AAG GTG ACG TTA TGG TT-3'
	Rv: 5'-TCA ATG TGA AAA GCC CAT CA-3'
CSLA9 (AT5G03760)	Fw: 5'-TCG GTT TAC TCG AAG GAG GA-3
	Rv: 5'-GAT TGC TTG TGC GAA AAG GT-3'
EXPANSIN A1 (AT1G69530)	Fw: 5'-AAG GCT ATG GAA CCA ACA CG-3
	Rv: 5'-ATT TTC GCC ACT GGA ATT G-3'
AHA1 (AT2G18960)	Fw: 5'-AAT TTT CGC CAC TGG AAT TG-3'
	Rv: 5'-ACC AAC TCC TTG ACC TGG TG-3'
AHA2 (AT4G30190)	Fw: 5'-TGC TCA AAG GAC ACT TCA CG-3'
	Rv: 5'GCC CTT TAG CTT CAC GAC TG-3'
CYCD3:1 (AT4G34160)	Fw: 5'-CCT CTC TGT AAT CTC CGA TTC AA-3'
	Rv: 5'- AAA GGG TTT GCA TCA ATC ACG-3'
BES1 (AT1G19350)	Fw: 5'- CGA GTT GGT GAT CCA CAC AAT A-3'
	Rv: 5'-GGA GGA AGC GAT GAA GGA ATA C -3'
DWF4 (AT3G50660)	Fw: 5'- GTG GGT GGA AAG TGT TAC CG-3
	Rv: 5 -CTG TTG CCA TCT CCA AGG AT -3 '
CPD (AT5G05690)	Fw: 5'-AGC AAC TCG GTA ACG ACA GG-3'
	Rv: 5'- CAG AGA GTG CAA CCC TAG CC-3

Supplemental Table 4. One-way ANOVA Analysis of RT-qPCR data utilizing Excell for RT-qPCR expression data in Control Conditions P value threshold set at 0.05.

AHA1

Anova: Single Factor

SUMMARY

Groups	*Count*	*Sum*	*Average*	*Variance*
Col-0	3	3,08793661	1,0293122	0,08158929
ttl1	3	1,21384146	0,40461382	0,01006205

ANOVA

Source of Variation	*SS*	*df*	*MS*	*F*	*P-value*	*F crit*
Between Groups	0,58537211	1	0,58537211	12,7738904	0,02329105	7,70864742
Within Groups	0,18330269	4	0,04582567			
Total	0,76867479	5				

AHA2

Anova: Single Factor

SUMMARY

Groups	*Count*	*Sum*	*Average*	*Variance*
Col-0	3	3,106764	1,035588	0,12241669
ttl1	3	0,75143775	0,25047925	0,00311039

ANOVA

Source of Variation	*SS*	*df*	*MS*	*F*	*P-value*	*F crit*
Between Groups	0,92459362	1	0,92459362	14,7313811	0,01848878	7,70864742
Within Groups	0,25105416	4	0,06276354			

Total	1,17564778	5				

BES1

Anova: Single Factor

SUMMARY

Groups	*Count*	*Sum*	*Average*	*Variance*
Col-0	2	1,6890286	0,8445143	0,00042008
ttl1	2	1,62164906	0,81082453	0,00067814

ANOVA

Source of Variation	*SS*	*df*	*MS*	*F*	*P-value*	*F crit*
Between Groups	0,001135	1	0,001135	2,06698125	0,28709414	18,5128205
Within Groups	0,00109822	2	0,00054911			
Total	0,00223322	3				

CESA1

Anova: Single Factor

SUMMARY

Groups	*Count*	*Sum*	*Average*	*Variance*
Col-0	*3*	*3,02021514*	*1,00673838*	*0,0193292*
ttl1	*3*	*1,55438591*	*0,51812864*	*0,01400333*

ANOVA

Source of Variation	*SS*	*df*	*MS*	*F*	*P-value*	*F crit*
Between Groups	0,35810922	1	0,35810922	21,4870704	0,00976749	7,70864742
Within Groups	0,06666506	4	0,01666627			
Total	0,42477428	5				

CESA3

Anova: Single Factor

SUMMARY

Groups	*Count*	*Sum*	*Average*	*Variance*

Col-0	3	3,04324076	1,01441359	0,04341288
ttl1	3	2,54317742	0,84772581	0,08493571

ANOVA

Source of Variation	*SS*	*df*	*MS*	*F*	*P-value*	*F crit*
Between Groups	0,04167722	1	0,04167722	0,64943795	0,46549338	7,70864742
Within Groups	0,25669719	4	0,0641743			
Total	0,29837442	5				

CESA6

Anova: Single Factor

SUMMARY

Groups	*Count*	*Sum*	*Average*	*Variance*
Col-0	3	3,02844704	1,00948235	0,02862059
ttl1	3	1,3998204	0,4666068	0,00939779

ANOVA

Source of Variation	*SS*	*df*	*MS*	*F*	*P-value*	*F crit*
Between Groups	0,44207079	1	0,44207079	23,2556326	0,0085081	7,70864742
Within Groups	0,07603677	4	0,01900919			
Total	0,51810756	5				

CSLA9

Anova: Single Factor

SUMMARY

Groups	*Count*	*Sum*	*Average*	*Variance*
Col-0	3	3,0595446	1,0198482	0,06582644
ttl1	3	1,91480552	0,63826851	0,01706182

ANOVA

Source of Variation	*SS*	*df*	*MS*	*F*	*P-value*	*F crit*
Between Groups	0,21840459	1	0,21840459	5,26985584	0,08334005	7,70864742

Within Groups	0,16577652	4	0,04144413			
Total	0,38418112	5				

COBRA

Anova: Single Factor

SUMMARY

Groups	*Count*	*Sum*	*Average*	*Variance*
Col-0	2	1,99051563	0,99525782	0,00033018
ttl1	2	0,9528354	0,4764177	0,00560215

ANOVA

Source of Variation	*SS*	*df*	*MS*	*F*	*P-value*	*F crit*
Between Groups	0,26919506	1	0,26919506	90,7552401	0,01083981	18,5128205
Within Groups	0,00593233	2	0,00296617			
Total	0,27512739	3				

CPD

Anova: Single Factor

SUMMARY

Groups	*Count*	*Sum*	*Average*	*Variance*
Col-0	3	3,00316012	1,00105337	0,00318405
ttl1	3	2,06363494	0,68787831	0,00656861

ANOVA

Source of Variation	*SS*	*df*	*MS*	*F*	*P-value*	*F crit*
Between Groups	0,14711793	1	0,14711793	30,1698142	0,0053537	7,70864742
Within Groups	0,01950531	4	0,00487633			
Total	0,16662324	5				

CYCD3;1

Anova: Single Factor

SUMMARY

Groups	*Count*	*Sum*	*Average*	*Variance*
Col-0	3	3,01472769	1,00490923	0,0153924
ttl1	3	1,77698094	0,59232698	0,00626806

ANOVA

Source of Variation	*SS*	*df*	*MS*	*F*	*P-value*	*F crit*
Between Groups	0,25533617	1	0,25533617	23,5762504	0,00830606	7,70864742
Within Groups	0,04332091	4	0,01083023			
Total	0,29865708	5				

DWF4

Anova: Single Factor

SUMMARY

Groups	*Count*	*Sum*	*Average*	*Variance*
Col-0	3	3,06292863	1,02097621	0,06990463
ttl1	3	1,18859848	0,39619949	0,00515324

ANOVA

Source of Variation	*SS*	*df*	*MS*	*F*	*P-value*	*F crit*
Between Groups	0,58551892	1	0,58551892	15,6017998	0,01681926	7,70864742
Within Groups	0,15011574	4	0,03752893			
Total	0,73563466	5				

EXPA1

Anova: Single Factor

SUMMARY

Groups	*Count*	*Sum*	*Average*	*Variance*
Col-0	3	3,04672516	1,01557505	0,04849267
ttl1	3	3,24890585	1,08296862	0,07491119

ANOVA

Source of Variation	SS	df	MS	F	P-value	F crit
Between Groups	0,00681284	1	0,00681284	0,11041533	0,75635536	7,70864742
Within Groups	0,24680771	4	0,06170193			
Total	0,25362055	5				

TTL3

Anova: Single Factor

SUMMARY

Groups	*Count*	*Sum*	*Average*	*Variance*
Col-0	2	1,99429909	0,99714954	0,01045787
ttl1	2	1,81119124	0,90559562	0,07766462

ANOVA

Source of Variation	*SS*	*df*	*MS*	*F*	*P-value*	*F crit*
Between Groups	0,00838212	1	0,00838212	0,19023795	0,70528454	18,5128205
Within Groups	0,08812249	2	0,04406125			
Total	0,09650461	3				

Supplemental Table 5. ANOVA Analysis of RT-qPCR data utilizing INFOSTAT with a multiple testing corrected P value threshold set at 0.05.

New table : 2/5/2024 - 4:56:51 PM - [Version : 4/30/2020]

Gen	Variable	N	R^2	Adj R^2	CV
AHA1	lfc	18	0.91	0.87	13.42

Analysis of variance table (Partial SS)

S.V.	SS	df	MS	F	p-value
Model	3.13	5	0.63	24.08	<0.0001
Condicion	2.38	2	1.19	45.83	<0.0001
Genotipo	0.01	1	0.01	0.51	0.4902
Condicion*Genotipo	0.73	2	0.37	14.11	0.0007
Error	0.31	12	0.03		
Total	3.44	17			

Test:Tukey Alpha:=0.05 LSD:=0.24826

Error: 0.0260 df: 12

Condicion	Means	n	S.E.

Control	0.72	6	0.07	A
shock	1.29	6	0.07	B
gradiente	1.59	6	0.07	C

Means with a common letter are not significantly different (p > 0.05)

Test:Tukey Alpha:=0.05 LSD:=0.16554

Error: 0.0260 df: 12

Genotipo	Means	n	S.E.	
ttl1	1.17	9	0.05	A
Col-0	1.23	9	0.05	A

Means with a common letter are not significantly different (p > 0.05)

Test:Tukey Alpha:=0.05 LSD:=0.44203

Error: 0.0260 df: 12

Condicion	Genotipo	Means	n	S.E.				
Control	ttl1	0.40	3	0.09	A			
Control	Col-0	1.03	3	0.09		B		
shock	Col-0	1.18	3	0.09		B	C	
shock	ttl1	1.40	3	0.09		B	C	D
gradiente	Col-0	1.47	3	0.09			C	D
gradiente	ttl1	1.72	3	0.09				D

Means with a common letter are not significantly different (p > 0.05)

Gen	Variable	N	R^2	Adj R^2	CV
AHA2	lfc	18	0.93	0.91	17.65

Analysis of variance table (Partial SS)

S.V.	SS	df	MS	F	p-value
Model	4.80	5	0.96	33.57	<0.0001
Condicion	3.72	2	1.86	65.08	<0.0001
Genotipo	0.11	1	0.11	3.73	0.0773
Condicion*Genotipo	0.97	2	0.49	16.98	0.0003
Error	0.34	12	0.03		
Total	5.14	17			

Test:Tukey Alpha:=0.05 LSD:=0.26035

Error: 0.0286 df: 12

Condicion	Means	n	S.E.		
shock	0.63	6	0.07	A	
Control	0.64	6	0.07	A	
gradiente	1.60	6	0.07		B

Means with a common letter are not significantly different (p > 0.05)

Test:Tukey Alpha:=0.05 LSD:=0.17361

Error: 0.0286 df: 12

Genotipo	Means	n	S.E.	
ttl1	0.88	9	0.06	A
Col-0	1.03	9	0.06	A

Means with a common letter are not significantly different (p > 0.05)

Test:Tukey Alpha:=0.05 LSD:=0.46357

Error: 0.0286 df: 12

Condicion	Genotipo	Means	n	S.E.				
Control	ttl1	0.25	3	0.10	A			
shock	Col-0	0.47	3	0.10	A	B		
shock	ttl1	0.79	3	0.10		B	C	
Control	Col-0	1.04	3	0.10			C	
gradiente	Col-0	1.60	3	0.10				D
gradiente	ttl1	1.60	3	0.10				D

Means with a common letter are not significantly different (p > 0.05)

Gen	Variable	N	R^2	Adj R^2	CV
BES1	lfc	18	0.67	0.53	21.85

Analysis of variance table (Partial SS)

S.V.	SS	df	MS	F	p-value
Model	0.73	5	0.15	4.90	0.0113
Condicion	0.67	2	0.34	11.27	0.0018
Genotipo	0.02	1	0.02	0.60	0.4539
Condicion*Genotipo	0.04	2	0.02	0.67	0.5297
Error	0.36	12	0.03		
Total	1.09	17			

Test:Tukey Alpha:=0.05 LSD:=0.26651

Error: 0.0299 df: 12

Condicion	Means	n	S.E.		
gradiente	0.52	6	0.07	A	
shock	0.91	6	0.07		B
control	0.94	6	0.07		B

Means with a common letter are not significantly different (p > 0.05)

Test:Tukey Alpha:=0.05 LSD:=0.17772

Error: 0.0299 df: 12

Genotipo	Means	n	S.E.	
ttl1	0.76	9	0.06	A
Col-0	0.82	9	0.06	A

Means with a common letter are not significantly different (p > 0.05)

Test:Tukey Alpha:=0.05 LSD:=0.47454

Error: 0.0299 df: 12

Condicion	Genotipo	Means	n	S.E.		
gradiente	ttl1	0.48	3	0.10	A	
gradiente	Col-0	0.55	3	0.10	A	
control	ttl1	0.85	3	0.10	A	B
shock	Col-0	0.89	3	0.10	A	B
shock	ttl1	0.94	3	0.10	A	B
control	Col-0	1.03	3	0.10		B

Means with a common letter are not significantly different (p > 0.05)

Gen	Variable	N	R^2	Adj R^2	CV
CESA1	lfc	18	0.63	0.48	21.67

Analysis of variance table (Partial SS)

S.V.	SS	df	MS	F	p-value

Model	0.82	5	0.16	4.14	0.0203
Condicion	0.34	2	0.17	4.26	0.0400
Genotipo	0.03	1	0.03	0.83	0.3794
Condicion*Genotipo	0.45	2	0.22	5.68	0.0184
Error	0.47	12	0.04		
Total	1.29	17			

Test:Tukey Alpha:=0.05 LSD:=0.30605

Error: 0.0395 df: 12

Condicion	Means	n	S.E.		
Control	0.76	6	0.08	A	
shock	0.89	6	0.08	A	B
gradiente	1.09	6	0.08		B

Means with a common letter are not significantly different (p > 0.05)

Test:Tukey Alpha:=0.05 LSD:=0.20408

Error: 0.0395 df: 12

Genotipo	Means	n	S.E.	
ttl1	0.87	9	0.07	A
Col-0	0.96	9	0.07	A

Means with a common letter are not significantly different (p > 0.05)

Test:Tukey Alpha:=0.05 LSD:=0.54493

Error: 0.0395 df: 12

Condicion	Genotipo	Means	n	S.E.		
Control	ttl1	0.52	3	0.11	A	
shock	Col-0	0.75	3	0.11	A	B
Control	Col-0	1.01	3	0.11	A	B
shock	ttl1	1.03	3	0.11	A	B
gradiente	ttl1	1.07	3	0.11		B
gradiente	Col-0	1.12	3	0.11		B

Means with a common letter are not significantly different (p > 0.05)

Gen	Variable	N	R^2	Adj R^2	CV
CESA3	lfc	18	0.66	0.52	32.92

Analysis of variance table (Partial SS)

S.V.	SS	df	MS	F	p-value
Model	4.26	5	0.85	4.63	0.0138
Condicion	1.71	2	0.85	4.64	0.0321
Genotipo	1.24	1	1.24	6.74	0.0234
Condicion*Genotipo	1.31	2	0.65	3.56	0.0610
Error	2.21	12	0.18		
Total	6.46	17			

Test:Tukey Alpha:=0.05 LSD:=0.66028

Error: 0.1838 df: 12

Condicion	Means	n	S.E.		
Control	0.93	6	0.18	A	
shock	1.29	6	0.18	A	B
gradiente	1.69	6	0.18		B

Means with a common letter are not significantly different (p > 0.05)

Test:Tukey Alpha:=0.05 LSD:=0.44029

Error: 0.1838 df: 12

Genotipo	Means	n	S.E.	
Col-0	1.04	9	0.14	A
ttl1	1.56	9	0.14	B

Means with a common letter are not significantly different (p > 0.05)

Test:Tukey Alpha:=0.05 LSD:=1.17566

Error: 0.1838 df: 12

Condicion	Genotipo	Means	n	S.E.		
Control	ttl1	0.85	3	0.25	A	
shock	Col-0	0.99	3	0.25	A	
Control	Col-0	1.01	3	0.25	A	
gradiente	Col-0	1.11	3	0.25	A	B
shock	ttl1	1.59	3	0.25	A	B
gradiente	ttl1	2.26	3	0.25		B

Means with a common letter are not significantly different (p > 0.05)

Gen	Variable	N	R^2	Adj R^2	CV
CESA6	lfc	18	0.77	0.67	25.59

Analysis of variance table (Partial SS)

S.V.	SS	df	MS	F	p-value
Model	1.68	5	0.34	7.89	0.0017
Condicion	0.98	2	0.49	11.46	0.0016
Genotipo	1.2E-03	1	1.2E-03	0.03	0.8705
Condicion*Genotipo	0.70	2	0.35	8.24	0.0056
Error	0.51	12	0.04		
Total	2.20	17			

Test:Tukey Alpha:=0.05 LSD:=0.31824

Error: 0.0427 df: 12

Condicion	Means	n	S.E.		
shock	0.56	6	0.08	A	
Control	0.74	6	0.08	A	
gradiente	1.12	6	0.08		B

Means with a common letter are not significantly different (p > 0.05)

Test:Tukey Alpha:=0.05 LSD:=0.21221

Error: 0.0427 df: 12

Genotipo	Means	n	S.E.	
Col-0	0.80	9	0.07	A
ttl1	0.82	9	0.07	A

Means with a common letter are not significantly different (p > 0.05)

Test:Tukey Alpha:=0.05 LSD:=0.56663

Error: 0.0427 df: 12

Condicion	Genotipo	Means	n	S.E.			
shock	Col-0	0.41	3	0.12	A		
Control	ttl1	0.47	3	0.12	A	B	
shock	ttl1	0.71	3	0.12	A	B	C
gradiente	Col-0	0.97	3	0.12	A	B	C
Control	Col-0	1.01	3	0.12		B	C

gradiente	ttl1	1.27	3	0.12	C

Means with a common letter are not significantly different (p > 0.05)

Gen	Variable	N	R^2	Adj R^2	CV
CESA9	lfc	18	0.57	0.39	24.85

Analysis of variance table (Partial SS)

S.V.	SS	df	MS	F	p-value
Model	0.54	5	0.11	3.15	0.0480
Condicion	0.10	2	0.05	1.46	0.2714
Genotipo	0.03	1	0.03	0.75	0.4040
Condicion*Genotipo	0.41	2	0.21	6.04	0.0153
Error	0.41	12	0.03		
Total	0.94	17			

Test:Tukey Alpha:=0.05 LSD:=0.28393

Error: 0.0340 df: 12

Condicion	Means	n	S.E.	
shock	0.65	6	0.08	A
gradiente	0.75	6	0.08	A
Control	0.83	6	0.08	A

Means with a common letter are not significantly different (p > 0.05)

Test:Tukey Alpha:=0.05 LSD:=0.18933

Error: 0.0340 df: 12

Genotipo	Means	n	S.E.	
ttl1	0.70	9	0.06	A
Col-0	0.78	9	0.06	A

Means with a common letter are not significantly different (p > 0.05)

Test:Tukey Alpha:=0.05 LSD:=0.50554

Error: 0.0340 df: 12

Condicion	Genotipo	Means	n	S.E.		
shock	Col-0	0.48	3	0.11	A	
Control	ttl1	0.64	3	0.11	A	B
gradiente	ttl1	0.66	3	0.11	A	B
shock	ttl1	0.82	3	0.11	A	B
gradiente	Col-0	0.84	3	0.11	A	B
Control	Col-0	1.02	3	0.11		B

Means with a common letter are not significantly different (p > 0.05)

Gen	Variable	N	R^2	Adj R^2	CV
COBRA	lfc	18	0.79	0.70	15.42

Analysis of variance table (Partial SS)

S.V.	SS	df	MS	F	p-value
Model	0.56	5	0.11	8.89	0.0010
Condicion	0.04	2	0.02	1.56	0.2493
Genotipo	0.06	1	0.06	4.84	0.0482
Condicion*Genotipo	0.46	2	0.23	18.23	0.0002
Error	0.15	12	0.01		
Total	0.71	17			

Test:Tukey Alpha:=0.05 LSD:=0.17269

Error: 0.0126 df: 12

Condicion	Means	n	S.E.	
gradiente	0.66	6	0.05	A
control	0.76	6	0.05	A
shock	0.76	6	0.05	A

Means with a common letter are not significantly different (p > 0.05)

Test:Tukey Alpha:=0.05 LSD:=0.11515

Error: 0.0126 df: 12

Genotipo	Means	n	S.E.		
ttl1	0.67	9	0.04	A	
Col-0	0.79	9	0.04		B

Means with a common letter are not significantly different (p > 0.05)

Test:Tukey Alpha:=0.05 LSD:=0.30748

Error: 0.0126 df: 12

Condicion	Genotipo	Means	n	S.E.			
control	ttl1	0.52	3	0.06	A		
gradiente	ttl1	0.58	3	0.06	A		
shock	Col-0	0.61	3	0.06	A	B	
gradiente	Col-0	0.74	3	0.06	A	B	C
shock	ttl1	0.91	3	0.06		B	C
control	Col-0	1.00	3	0.06			C

Means with a common letter are not significantly different (p > 0.05)

Gen	Variable	N	R^2	Adj R^2	CV
CPD	lfc	18	0.84	0.78	11.50

Analysis of variance table (Partial SS)

S.V.	SS	df	MS	F	p-value
Model	0.41	5	0.08	12.85	0.0002
Condicion	0.22	2	0.11	17.44	0.0003
Genotipo	0.13	1	0.13	20.61	0.0007
Condicion*Genotipo	0.06	2	0.03	4.38	0.0373
Error	0.08	12	0.01		
Total	0.49	17			

Test:Tukey Alpha:=0.05 LSD:=0.12308

Error: 0.0064 df: 12

Condicion	Means	n	S.E.		
gradiente	0.58	6	0.03	A	
shock	0.66	6	0.03	A	
Control	0.84	6	0.03		B

Means with a common letter are not significantly different (p > 0.05)

Test:Tukey Alpha:=0.05 LSD:=0.08207

Error: 0.0064 df: 12

Genotipo	Means	n	S.E.		
ttl1	0.61	9	0.03	A	
Col-0	0.78	9	0.03		B

Means with a common letter are not significantly different (p > 0.05)

Test:Tukey Alpha:=0.05 LSD:=0.21915

Error: 0.0064 df: 12

Condicion	Genotipo	Means	n	S.E.		
gradiente	ttl1	0.50	3	0.05	A	
shock	ttl1	0.64	3	0.05	A	
gradiente	Col-0	0.66	3	0.05	A	
shock	Col-0	0.68	3	0.05	A	
Control	ttl1	0.69	3	0.05	A	
Control	Col-0	1.00	3	0.05		B

Means with a common letter are not significantly different (p > 0.05)

Gen	Variable	N	R^2	Adj R^2	CV
CYCD3;1	lfc	18	0.83	0.75	30.31

Analysis of variance table (Partial SS)

S.V.	SS	df	MS	F	p-value
Model	20.77	5	4.15	11.37	0.0003
Condicion	18.56	2	9.28	25.40	<0.0001
Genotipo	1.47	1	1.47	4.04	0.0676
Condicion*Genotipo	0.73	2	0.37	1.00	0.3951
Error	4.39	12	0.37		
Total	25.16	17			

Test:Tukey Alpha:=0.05 LSD:=0.93120

Error: 0.3655 df: 12

Condicion	Means	n	S.E.			
Control	0.80	6	0.25	A		
shock	1.90	6	0.25		B	
gradiente	3.28	6	0.25			C

Means with a common letter are not significantly different (p > 0.05)

Test:Tukey Alpha:=0.05 LSD:=0.62095

Error: 0.3655 df: 12

Genotipo	Means	n	S.E.	
ttl1	1.71	9	0.20	A
Col-0	2.28	9	0.20	A

Means with a common letter are not significantly different (p > 0.05)

Test:Tukey Alpha:=0.05 LSD:=1.65804

Error: 0.3655 df: 12

Condicion	Genotipo	Means	n	S.E.			
Control	ttl1	0.59	3	0.35	A		
Control	Col-0	1.00	3	0.35	A		
shock	ttl1	1.81	3	0.35	A	B	
shock	Col-0	1.99	3	0.35	A	B	
gradiente	ttl1	2.72	3	0.35		B	C
gradiente	Col-0	3.84	3	0.35			C

Means with a common letter are not significantly different (p > 0.05)

Gen	Variable	N	R^2	Adj R^2	CV

DWF4	lfc	18	0.86	0.80	28.67

Analysis of variance table (Partial SS)

S.V.	SS	df	MS	F	p-value
Model	1.21	5	0.24	14.31	0.0001
Condicion	0.61	2	0.31	18.07	0.0002
Genotipo	0.22	1	0.22	12.71	0.0039
Condicion*Genotipo	0.38	2	0.19	11.34	0.0017
Error	0.20	12	0.02		
Total	1.41	17			

Test:Tukey Alpha:=0.05 LSD:=0.20047

Error: 0.0169 df: 12

Condicion	Means	n	S.E.		
gradiente	0.28	6	0.05	A	
shock	0.38	6	0.05	A	
Control	0.71	6	0.05		B

Means with a common letter are not significantly different (p > 0.05)

Test:Tukey Alpha:=0.05 LSD:=0.13368

Error: 0.0169 df: 12

Genotipo	Means	n	S.E.		
ttl1	0.34	9	0.04	A	
Col-0	0.56	9	0.04		B

Means with a common letter are not significantly different (p > 0.05)

Test:Tukey Alpha:=0.05 LSD:=0.35694

Error: 0.0169 df: 12

Condicion	Genotipo	Means	n	S.E.		
gradiente	ttl1	0.24	3	0.08	A	
gradiente	Col-0	0.32	3	0.08	A	
shock	Col-0	0.35	3	0.08	A	
Control	ttl1	0.40	3	0.08	A	
shock	ttl1	0.40	3	0.08	A	
Control	Col-0	1.02	3	0.08		B

Means with a common letter are not significantly different (p > 0.05)

Gen	Variable	N	R^2	Adj R^2	CV
EXPA1	lfc	18	0.84	0.77	15.81

Analysis of variance table (Partial SS)

S.V.	SS	df	MS	F	p-value
Model	2.80	5	0.56	12.26	0.0002
Condicion	1.39	2	0.69	15.15	0.0005
Genotipo	0.81	1	0.81	17.81	0.0012
Condicion*Genotipo	0.60	2	0.30	6.58	0.0118
Error	0.55	12	0.05		
Total	3.35	17			

Test:Tukey Alpha:=0.05 LSD:=0.32927

Error: 0.0457 df: 12

Condicion	Means	n	S.E.	
Control	1.05	6	0.09	A

gradiente	1.29	6	0.09	A
shock	1.72	6	0.09	B

Means with a common letter are not significantly different (p > 0.05)

Test:Tukey Alpha:=0.05 LSD:=0.21957

Error: 0.0457 df: 12

Genotipo	Means	n	S.E.	
Col-0	1.14	9	0.07	A
ttl1	1.57	9	0.07	B

Means with a common letter are not significantly different (p > 0.05)

Test:Tukey Alpha:=0.05 LSD:=0.58629

Error: 0.0457 df: 12

Condicion	Genotipo	Means	n	S.E.		
Control	Col-0	1.02	3	0.12	A	
Control	ttl1	1.08	3	0.12	A	
gradiente	Col-0	1.15	3	0.12	A	
shock	Col-0	1.26	3	0.12	A	
gradiente	ttl1	1.43	3	0.12	A	
shock	ttl1	2.18	3	0.12		B

Means with a common letter are not significantly different (p > 0.05)

Gen	Variable	N	R^2	Adj R^2	CV
Pectato liasa	lfc	18	0.81	0.72	19.21

Analysis of variance table (Partial SS)

S.V.	SS	df	MS	F	p-value
Model	1.95	5	0.39	9.94	0.0006
Condicion	1.70	2	0.85	21.54	0.0001
Genotipo	4.3E-03	1	4.3E-03	0.11	0.7457
Condicion*Genotipo	0.26	2	0.13	3.24	0.0749
Error	0.47	12	0.04		
Total	2.43	17			

Test:Tukey Alpha:=0.05 LSD:=0.30554

Error: 0.0393 df: 12

Condicion	Means	n	S.E.		
shock	0.77	6	0.08	A	
control	0.86	6	0.08	A	
gradiente	1.46	6	0.08		B

Means with a common letter are not significantly different (p > 0.05)

Test:Tukey Alpha:=0.05 LSD:=0.20374

Error: 0.0393 df: 12

Genotipo	Means	n	S.E.	
Col-0	1.02	9	0.07	A
ttl1	1.05	9	0.07	A

Means with a common letter are not significantly different (p > 0.05)

Test:Tukey Alpha:=0.05 LSD:=0.54403

Error: 0.0393 df: 12

Condicion	Genotipo	Means	n	S.E.

shock	Col-0	0.70	3	0.11	A
control	ttl1	0.71	3	0.11	A
shock	ttl1	0.85	3	0.11	A B
control	Col-0	1.01	3	0.11	A B
gradiente	Col-0	1.34	3	0.11	B C
gradiente	ttl1	1.59	3	0.11	C

Means with a common letter are not significantly different (p > 0.05)

Gen	Variable	N	R^2	Adj R^2	CV
TTL1	lfc	9	nd	0.42	20.44

Analysis of variance table (Partial SS)

S.V.	SS	df	MS	F	p-value
Model	0.22	2	0.11	3.90	0.0822
Condicion	0.22	2	0.11	3.90	0.0822
Genotipo	0.00	0	0.00	nd	nd
Condicion*Genotipo	0.00	0	0.00	nd	nd
Error	0.17	6	0.03		
Total	0.39	8			

Test:Tukey Alpha:=0.05 LSD:=0.41962

Error: 0.0281 df: 6

Condicion	Means	n	S.E.	
shock	0.63	3	1.7480142918471E45	A
gradiente	0.82	3	1.71640702665233E30	A
control	1.01	3	1.7480142918471E45	A

Means with a common letter are not significantly different (p > 0.05)

Test:Tukey Alpha:=0.05 LSD:=0.41962

Error: 0.0281 df: 6

Genotipo	Means	n	S.E.	
Col-0	0.82	9	5.17516190448665E29	A

Means with a common letter are not significantly different (p > 0.05)

Test:Tukey Alpha:=0.05 LSD:=0.41962

Error: 0.0281 df: 6

Condicion	Genotipo	Means	n	S.E.	
shock	Col-0	0.63	3	1.7480142918471E45	A
gradiente	Col-0	0.82	3	1.71640702665233E30	A
control	Col-0	1.01	3	1.7480142918471E45	A

Means with a common letter are not significantly different (p > 0.05)

Gen	Variable	N	R^2	Adj R^2	CV
TTL3	lfc	18	0.85	0.78	15.63

Analysis of variance table (Partial SS)

S.V.	SS	df	MS	F	p-value
Model	2.93	5	0.59	13.20	0.0002
Condicion	2.36	2	1.18	26.62	<0.0001
Genotipo	1.4E-03	1	1.4E-03	0.03	0.8613
Condicion*Genotipo	0.57	2	0.28	6.37	0.0130
Error	0.53	12	0.04		

Total	3.46	17

Test:Tukey Alpha:=0.05 LSD:=0.32453

Error: 0.0444 df: 12

Condicion	Means	n	S.E.			
Control	0.93	6	0.09	A		
shock	1.31	6	0.09		B	
gradiente	1.81	6	0.09			C

Means with a common letter are not significantly different (p > 0.05)

Test:Tukey Alpha:=0.05 LSD:=0.21640

Error: 0.0444 df: 12

Genotipo	Means	n	S.E.	
Col-0	1.34	9	0.07	A
ttl1	1.36	9	0.07	A

Means with a common letter are not significantly different (p > 0.05)

Test:Tukey Alpha:=0.05 LSD:=0.57784

Error: 0.0444 df: 12

Condicion	Genotipo	Means	n	S.E.			
Control	ttl1	0.85	3	0.12	A		
Control	Col-0	1.00	3	0.12	A	B	
shock	Col-0	1.05	3	0.12	A	B	
shock	ttl1	1.57	3	0.12		B	C
gradiente	ttl1	1.66	3	0.12			C
gradiente	Col-0	1.96	3	0.12			C

Means with a common letter are not significantly different (p > 0.05)

Gen	Variable	N	R^2	Adj R^2	CV
TTL4	lfc	18	0.55	0.36	38.01

Analysis of variance table (Partial SS)

S.V.	SS	df	MS	F	p-value
Model	28.88	5	5.78	2.95	0.0577
Condicion	21.24	2	10.62	5.43	0.0209
Genotipo	6.43	1	6.43	3.29	0.0949
Condicion*Genotipo	1.22	2	0.61	0.31	0.7385
Error	23.47	12	1.96		
Total	52.35	17			

Test:Tukey Alpha:=0.05 LSD:=2.15402

Error: 1.9557 df: 12

Condicion	Means	n	S.E.		
control	2.67	6	0.57	A	
gradiente	3.19	6	0.57	A	B
shock	5.19	6	0.57		B

Means with a common letter are not significantly different (p > 0.05)

Test:Tukey Alpha:=0.05 LSD:=1.43635

Error: 1.9557 df: 12

Genotipo	Means	n	S.E.	
Col-0	3.08	9	0.47	A
ttl1	4.28	9	0.47	A

Means with a common letter are not significantly different (p > 0.05)

Test:Tukey Alpha:=0.05 LSD:=3.83531

Error: 1.9557 df: 12

Condicion	Genotipo	Means	n	S.E.		
control	Col-0	2.11	3	0.81	A	
gradiente	Col-0	2.89	3	0.81	A	B
control	ttl1	3.23	3	0.81	A	B
gradiente	ttl1	3.48	3	0.81	A	B
shock	Col-0	4.25	3	0.81	A	B
shock	ttl1	6.12	3	0.81		B

Means with a common letter are not significantly different (p > 0.05)
